# Developing the Active Communities Tool to Implement the Community Guide’s Built Environment Recommendation for Increasing Physical Activity

**DOI:** 10.5888/pcd17.200118

**Published:** 2020-11-12

**Authors:** Kelly R. Evenson, Anna K. Porter, Kristine L. Day, Carol McPhillips-Tangum, Karma E. Harris, Chris S. Kochtitzky, Philip Bors

**Affiliations:** 1 Department of Epidemiology, Gillings School of Global Public Health, University of North Carolina – Chapel Hill, Chapel Hill, North Carolina; 2 Center for Health Promotion and Disease Prevention, University of North Carolina – Chapel Hill, Chapel Hill, North Carolina; 3 College of Nursing and Health Professions, University of Southern Mississippi, Hattiesburg, Mississippi; 4 Division of Nutrition, Physical Activity, and Obesity; National Center for Chronic Disease Prevention and Health Promotion; Centers for Disease Control and Prevention, Atlanta, Georgia; 5 National Association of Chronic Disease Directors, Decatur, Georgia; 6 Healthy Places by Design, TSNE MissionWorks, Chapel Hill, North Carolina

## Abstract

Physical activity is higher in communities that include supportive features for walking and bicycling. In 2016, the Community Preventive Services Task Force released a systematic review of built environment approaches to increase physical activity. The results of the review recommended approaches that combine interventions to improve pedestrian and bicycle transportation systems with land use and environmental design strategies. Because the recommendation was multifaceted, the Centers for Disease Control and Prevention determined that communities could benefit from an assessment tool to address the breadth of the Task Force recommendations. The purpose of this article is to describe the systematic approach used to develop the Active Communities Tool. First, we created and refined a logic model and community theory of change for tool development. Second, we reviewed existing community-based tools and abstracted key elements (item domains, advantages, disadvantages, updates, costs, permissions to use, and psychometrics) from 42 tools. The review indicated that no tool encompassed the breadth of the Community Guide recommendations for communities. Third, we developed a new tool and pilot tested its use with 9 diverse teams with public health and planning expertise. Final revisions followed from pilot team and expert input. The Active Communities Tool comprises 6 modules addressing all 8 interventions recommended by the Task Force. The tool is designed to help cross-sector teams create an action plan for improving community built environments that promote physical activity and may help to monitor progress toward achieving community conditions known to promote physical activity.

**Summary**

**What is already known on this topic?**

The built environment influences physical activity through the pedestrian and bicycle transportation system (eg, activity-friendly destinations) and both land use and environmental design (eg, everyday destinations).

**What is added by this report?**

This report describes the process of developing the Active Communities Tool to implement the recommended built environment components that affect physical activity.

**What are the implications for public health practice?**

The Active Communities Tool, developed by using a sound protocol, is available for public use. The overarching process may be applicable in other public health settings; namely, to develop an assessment tool that can help community members select intervention targets and monitor changes over time.

## Introduction

Lack of physical activity is a leading contributor to death in the United States (1–3). One of the most important actions that people of all ages and abilities can take to improve their health is to be physically active (4). In the United States, walking is the most commonly reported leisure activity among adults (5,6). This may be because walking does not require special skills or equipment, and most people are able to walk (4). Bicycling is also a commonly reported physical activity; in 2012, 21% of the US adult population reported that they rode a bicycle in the past 30 days for any purpose (7). However, among US adults, the prevalence of no reported leisure-time physical activity remains high (25% in 2018) (8). Similar concerns abound among US youth, many of whom are not physically active (9).

When communities include supportive features for walking and bicycling, physical activity is higher (10). Barriers to walking and bicycling can be considered from a socioecologic perspective, identifying areas that affect behavior at the intrapersonal, interpersonal, institutional, policy, and environmental levels (11). By focusing on the policy and environmental level to support walking and bicycling, the built environment can offer safe and convenient access to parks, sidewalks, greenways, and bicycle lanes (12). Because the built environment can affect physical activity, and because walking is such a common type of activity, in 2015 the US Surgeon General released “A Call to Action to Promote Walking and Walkable Communities,” describing the urgent public health problem around walking (4). The call to action provided strategies that communities can use to support walking, resulting in long-lasting changes to improve the health of Americans.

Following this, in December 2016, the Community Preventive Services Task Force (Task Force) released a recommendation on built environment approaches to increase physical activity (13). The Task Force based its recommendation on a systematic review that included 16 longitudinal studies and 74 cross-sectional studies published through June 2014. The Task Force recommended combining 2 intervention approaches: (1) pedestrian and bicycle transportation system and (2) land use and environment design. Table 1 summarizes these approaches and provides examples. In the pedestrian, bicycle, and transit transportation systems, interventions focused on producing activity-friendly routes and included street pattern design and connectivity, pedestrian infrastructure, bicycle infrastructure, and public transit infrastructure and access. In land use and environmental design, interventions focused on increasing access to everyday destinations and included mixed land use, increased residential density, community or neighborhood proximity to destinations, and parks and recreational facility access.

**Table 1:** Recommended Interventions, Examples, and Corresponding Active Communities Tool (ACT) Module^a^

Approach	Intervention	Examples	ACT Module^b^
Pedestrian and bicycle transportation system interventions: activity-friendly routes	Street pattern design and connectivity	Designs that increase street connections and create both multiple route options and shorter block lengths	1. Street Design and Connectivity
	Pedestrian infrastructure^c^	Sidewalks, trails, traffic calming, intersection design, street lighting, and landscaping	2. Infrastructure to Accommodate Pedestrians and Bicyclists
	Bicycle infrastructure^c^	Bicycle systems, protected bicycle lanes, trails, traffic calming, intersection design, street lighting, and landscaping	
	Public transit infrastructure and access	Expanded transit service, times, locations, and connections	3. Public Transportation
Land use and environmental design interventions: everyday destinations	Mixed land use	Residential, commercial, cultural, institutional, or industrial land uses that are physically and functionally integrated to provide a complementary or balanced mix of restaurants, office buildings, housing, and shops	4. Land Use Planning
	Increased residential density	Smart growth communities and new urbanist designs, relaxed planning restrictions in appropriate locations to reduce sprawl, sustainable compact cities and communities with affordable housing	
	Proximity to community or neighborhood destinations	Community destinations such as stores, health facilities, banks, and social clubs that are accessible and close to each other	
	Parks and recreational facility access	Public parks, public recreational facilities, and private fitness facilities	5. Parks and Recreational Facilities
Schools^d^	Supportive plans, policies, built environment, and resources	Supports for walking or bicycling to and from school, wellness policies, and joint use agreements for shared use of school facilities for physical activity	6. Schools

a Active Communities Tool (ACT): an action planning guide and assessment modules to improve community built environments to promote physical activity (14). Part of this table is modified from tables 1 and 2 of the Community Guide recommendations (13), page 3.

b ACT modules can be accessed at https://www.cdc.gov/physicalactivity/community-strategies/active-communities-tool/assessment-modules.html (14).

c Examples of pedestrian and bicycle infrastructure can be found at http://www.pedbikeinfo.org/ (20).

d Schools were not a separate recommended intervention from the Community Guide, but were a separate ACT module that covered interventions relating to the 1) pedestrian and bicycle transportation system and 2) land use and environment design.

The Centers for Disease Control and Prevention, Division of Nutrition, Physical Activity, and Obesity (CDC) supports communities and states to implement the Community Guide built environment recommendation (13). Therefore, CDC supported the development of the Active Communities Tool (ACT) (14). The purpose of this article is to describe development, pilot testing, and refinement of ACT, which is intended to be used by community stakeholders to assess the condition of their built environment related to physical activity. We also highlight strengths and limitations of ACT as well as opportunities for its use in diverse communities across the United States.

## Development

The development of ACT followed a series of steps: convening an advisory panel, reviewing existing tools, diagramming how community changes occur, and mapping candidate items for the tool to those changes to develop a draft tool.

First, the authors convened an ad hoc national external advisory group to provide individual-level feedback on the development process (14). Advisory group members were chosen for their expertise in the built environment, transportation, and physical activity. The group provided feedback on a logic model and a community theory of change diagram to depict the process of change in communities to increase physical activity through recommended interventions.

Second, to inform development of ACT, we identified relevant tools through web and PubMed searches, and from those recommended by the research team, advisory group, and pilot teams. We conducted a detailed review of 42 community-based tools on the built environment and policy characteristics that might address any of the Community Guide recommended interventions. We sought existing tools that included the following characteristics: user-friendly questions (ie, avoided jargon); evidence for reliability, validity, and sensitivity to change; positive user experiences; and a format that could be easily programmed into a survey system. From these tools, we abstracted the following key elements where available: advantages, disadvantages, updates, costs, psychometrics, and candidate items. Although most reviewed tools focused on the community level, we also reviewed state-level tools with the intent of modifying them for communities. The review indicated that no tool encompassed the breadth of the Community Guide recommendations (13), and tools generally lacked evidence for reliability and validity.

Third, we developed a first draft of ACT that contained modules based on the 8 intervention categories in the Task Force recommendation (street pattern design and connectivity, pedestrian infrastructure, bicycle infrastructure, public transit infrastructure and access, mixed land use, increased residential density, proximity to community or neighborhood destinations, and parks and recreational facility access) (Table 1). The draft tool focused questions at the community level rather than asking questions about a specific street, street intersection, or cul-de-sac as other tools did. We then developed intervention theory of change diagrams for each intervention category that depicted how we envisioned how plans, policies, resources (funding, personnel, partnerships), and the built environment affected each intervention category to help us select items from existing tools and identify any missing content. We based the initial module content on a list of candidate items drawn from the tools identified by our review, which required modification for our purpose. We drafted questions to address gaps highlighted by the theory of change diagrams. For ease of use in communities, we kept questions on schools in a single module, although the module spanned multiple intervention areas. We defined key terms to assist users who may not be familiar with certain concepts.

Following the initial draft of ACT, we met with the external national advisory group to further develop the theory of change diagrams, to identify missing questions, and to receive feedback on the initial draft of the tool. On the basis of this process and advisory group recommendations, we further revised the tool from 8 to 6 modules for ease of use (Table 2). Specifically, the modules on mixed land use, proximity to community or neighborhood destinations, and increased residential density were combined into one module titled “land use planning.”

**Table 2:** Pilot Testing of the Active Communities Tool^a^, by Module

Modules	No. of Teams	Median Minutes (Range in Minutes)	Confidence of the Team, n^b^		
			Very	Somewhat	Not Very
Street design and connectivity	6	33 (25–90)	5	1	0
Infrastructure to accommodate pedestrians and bicyclists	5^a^	150 (20–225)	3	2	0
Public transportation	7	30 (15–150)	3	3	1
Land use planning	6	65 (10–120)	4	2	0
Parks and recreational facilities	4	38 (30–60)	2	2	0
Schools	4	30 (15–240)	1	2	1

a The Active Communities Tool consists of an action planning guide and assessment modules to improve community built environments to promote physical activity (14). Six teams assessed this module, but 1 team did not answer the questions on the time the module took or confidence in the team’s answers.

b This question asked, “How confident was the team in its ability to answer the questions in this module accurately?” Response options included very confident, somewhat confident, or not very confident.

## Pilot Testing and Tool Refinement

Following the development of ACT, we invited 9 teams to pilot test the tool based on their knowledge, interest, or past experience as part of multidisciplinary teams to promote access and opportunity for physical activity through community, transportation, and the built environment. The 9 teams had recent experience in built environment interventions, including 5 that reported prior work on Complete Streets, 9 on master planning, 8 on Safe Routes to School, and 8 on zoning.

In April 2018, a webinar introduced pilot teams to ACT and addressed questions about the pilot testing process. Teams then pilot-tested the tool. All teams completed descriptive information about their community. We asked half of the teams to complete the pedestrian, bicycle, and transit modules, and we asked the other half to complete the land use and environmental design modules (Table 2). Some teams chose to complete more than their assigned number of modules. We asked that all teams provide detailed feedback on specific items as well as the overall modules and tool. For the purposes of pilot testing, each module ended with questions designed to capture self-assessed confidence in the answers recorded, feedback on the items, and length of time the module took to complete.

Most teams used the tool to assess their own community, but a few teams working at state government agencies completed the tool to assess other communities with which they were less familiar. Seven teams assessed one municipality while the other 2 teams applied it to an entire county. The teams used the tool to assess the following locations: Bloomfield, Connecticut; city and county of Honolulu, Hawaii; Des Moines, Iowa; Duluth, Minnesota; Albuquerque, New Mexico; Tulsa, Oklahoma; Hamilton County, Tennessee; St. George City, Utah; and Tacoma, Washington. All teams included representatives from public health and planning; 8 of the 9 teams included a transit representative. In addition, some teams brought in other experts to complete the modules, such as a school representative for the school module or a parks representative for the parks and recreational facilities module. The median time it took pilot teams to complete each module ranged from 30 to 150 minutes (Table 2). Almost all teams were somewhat or very confident in completing the module; 2 teams that responded “not very confident” lacked a key representative to complete those modules (transportation and school).

By using the completed module data, we reviewed individual items that had missing responses, “do not know” responses, or multiple responses (when there should have only been one response) for indication of problems. We also reviewed items that lacked variability to assess whether the question should be retained. The detailed feedback provided at the end of each module provided clarifying remarks to revise items.

A final call with the pilot teams provided context around the questions that needed revision or questions that were missing. During this discussion, some members of pilot teams said that the definitions provided clarity of the terms in the modules and encouraged expanding the use of them. Some members viewed the experience as an opportunity to create new partnerships, because completing the tool required multidisciplinary expertise. Several members also said that completing the tool was educational for them, in that they better understood how certain environment and policy decisions the tool asked about might affect physical activity. For example, one pilot tester wrote, “In general, considering the questions asked in the document has been helpful, and they reveal to me possible metrics against which we can measure our work. It is also a helpful reminder that the differences in jurisdictions have impacts on the accessibility, creation, and maintenance of pedestrian and bike facilities, and it points to the need for policies and procedures that speak to each other across jurisdictional boundaries.”

The pilot teams provided actionable feedback in several critical areas. First, 2 pilot teams tried completing the assessment at a county level and found it challenging, because numerous municipalities were contained in the assessed county, with variations in policies and environments across jurisdictions. We added clarity to encourage users that wanted to complete the assessment for a county or region to do so separately for each of the municipalities located within the larger geographic area. Second, in some instances the yes/no questions made it impossible to capture important nuances. We therefore reviewed every question with only 2 response options and considered ways of expanding the options for improved sensitivity. Third, the feedback from several pilot teams revealed areas where regional variation in environments and contexts might not be sufficiently addressed by the tool, such as snow removal or water rights. However, to keep the modules at a reasonable length we did not expand further into these specialized topics that were not relevant in most communities.

We completed a second revision of the tool based on the feedback of the pilot teams and shared this revised tool with the pilot teams. We hosted a webinar for advisors to provide additional feedback on the tool and discussed how the final version could be used. Feedback from the advisory group and other experts led to further edits to the tool.

The final version of the ACT comprises 6 modules, as follows, that integrate questions about the 8 recommended intervention areas (Table 3):

**Table 3:** Final Version of the Active Communities Tool^a^

Modules	Number of Items	Example Questions by Module			
		Plans	Policies	Environment	Resources
Street design and connectivity	13	Street plan, connectivity goals	Zoning codes, subdivision regulations	None	Funding for connectivity
Infrastructure to accommodate pedestrians and bicyclists	24/24/37^b^	Vision Zero plan, Complete Streets plan	Shared-use path policies, traffic island policies, dog leash policies	Lighting on roads and paths, maps of shared-use paths	Fee-in-lieu to develop parks, funding for bicycle/pedestrian projects
Public transportation	28	Transit plan	Community policies on transit	Route service, transit infrastructure maintenance	Funding for walking and bicycle infrastructure facilities at transit stops
Land use planning	26	Land use plan, comprehensive plan	Zoning, policies encouraging mixed-use	Percent infill, downtown district	Financial incentives for mixed use, affordable housing, or healthy food retail in or near residential areas
Parks and recreational facilities	39	Master park plan	Park siting policies, requirements for parks	Park maintenance, acres of park land	Funding for park maintenance, developer incentives for preserving open space
Schools	26	Plan that focused on increasing opportunities to walk and bicycle to/from school	Wellness policy to promote walking and bicycling to/from school	None	School coordinator to focus on safe walking and bicycling to/from school

a Active Communities Tool (ACT): an action planning guide and assessment modules to improve community built environments to promote physical activity (14).

b The item counts align with infrastructure to accommodate pedestrians and bicyclists (section A), pedestrians (section B), and bicyclists (section C).

- Module 1 – Street design and connectivity
- Module 2 – Infrastructure to accommodate pedestrians and bicyclists
- Module 3 – Public transportation
- Module 4 – Land use planning
- Module 5 – Parks and recreational facilities
- Module 6 – Schools

In addition to the tool, ACT includes an action planning guide to facilitate its use and to improve community environments to promote physical activity.

## Characteristics and Future Use of the Tool

ACT is freely available to the public for download and use (14). The tool is unique in its community-focused design, specificity to the evidence base for physical activity, and its comprehensiveness. It can be used to provide an assessment, identify opportunities for improvement by highlighting gaps, and may be able to monitor progress over time. It can help users select and plan actions to improve community built environments, plans, policies, and resources to increase physical activity. Moreover, the tool can be tailored to specific contexts and community goals.

A companion document, titled “A Guide for Developing an Action Plan to Improve Community Built Environments that Promote Physical Activity,” informs the action planning process specific to improving community built environments for physical activity (14). The “how to” document includes:

- background on the development of ACT,
- examples of disciplines to engage in when using ACT,
- a description of key steps to completing ACT,
- information, including resources, on how to develop an action plan with ACT results, and
- resources to develop an evaluation and remain sustainable for the long term.

The tool is not a scorecard to rank communities. Rather, it is a way to assess current conditions and identify areas for action. Although promotions and programs are important components of community interventions to promote and increase physical activity, they are not addressed in these modules. The tool ascertains characteristics relevant to all intervention examples highlighted by the Community Guide (13), and detailed in Table 1, except for private fitness facilities, which can be more accurately obtained through a combination of business directories and on-the-ground verification (15).

The tool is also not a street-level audit. Instead, the tool focuses on the policies, plans, resources, and select community-level aspects of the built environment necessary to achieve community conditions known to promote physical activity, as described by the Community Guide (13). The tool does not capture critical steps in the policy process, such as agenda building, policy formulation, adoption, and evaluation. The tool also does not capture quality of facilities, an important component when considering equity, because of the length it would have added. When quality is important to capture, supplemental detailed assessments can be considered to assess quality of community facilities, such as those relating to parks, transit, walking, bicycling, and schools.

Although the tool is meant to be comprehensive, it could still miss important community-level policies not identified through the Community Guide (13). For example, an Australian team reviewed current state-level policies related to transportation and land use (16). Their approach helped identify policies for which metrics did not exist (eg, availability of bicycle racks, footpaths, design of public transit stops). This same approach applied to a specific community may reveal additional items important for the community but not found on the tool. Also of note, the intervention strategies from the Community Guide (and therefore the tool) could miss elements that are important for rural communities, because none of the reviewed studies focused solely on rural areas (13).

The process we used to develop, pilot, refine, and finalize ACT did not include testing the questions for validity. For example, to test validity of questions on the existence of specific plans and the content in those plans, the document could be found and checked. Similarly, the report of certain policies could be cross-checked against published community codes and ordinances. The validity of other questions would be much harder to document, such as how often a specific plan was consulted or whether the community uses best practices for design for people who walk, bicycle, or use transit. Internal reliability was also not tested, which could involve having 2 people knowledgeable about the community independently complete the assessment. Practitioners who follow our example for the development of a community-based tool are encouraged to include evidence for validity when meaningful, such as documenting verification of answers from published plans or policies.

Several future efforts could be applied to the tool. First, testing of the tool is needed to assess validity, reliability, and sensitivity to change over time. If psychometrically sound, it could be implemented as an evaluation tool that is used repeatedly to assess change over time. Future iterations of the tool could allow attachment of files, such as copies of policies and plans, to corroborate findings and provide some evidence of validity. Second, the revised ACT was not retested with communities. It would be beneficial to test ACT with a larger representative sample of communities. Third, the tool could be expanded to link to guidance for next steps in specific intervention areas. For example, if a community indicates that Complete Streets is not addressed in their planning documents, guidance on how to initiate action in their community and links to other resources could be prompted by their answers. Best practices and exemplary plans or policies identified by the tool could be linked and highlighted to assist others. Fourth, a scoring system could be developed to allow communities to benchmark against other communities of similar size and type (eg, urban, suburban, rural).

## Conclusion

ACT (14) addresses the 2 major intervention areas recommended by the Community Guide (13): 1) pedestrian and bicycle transportation system and 2) land use and environmental design. It also supports one of the evidence-based strategies in CDC’s Active People, Healthy Nation initiative (17) and addresses a Healthy People 2020 goal by addressing the social determinants of health such as physical environments that promote good health for all (18). The tool is most appropriate at the community level, such as for a municipality, and facilitates important connections between public health, planning, and transportation (19). ACT is designed so that communities can choose which modules to complete based on their intervention priorities. By using the tool, communities can assess local supports for physical activity and identify gaps to be addressed.
